# Supplementary material for: Patterns of Intron Gain and Loss in Fungi
Source: PLoS Biol. 2004 Nov 30;2(12):e422. doi: 10.1371/journal.pbio.0020422 (PMC532390; doi:10.1371/journal.pbio.0020422)
Supplement: Table S1 — Also available at http://genes.mit.edu/NielsenEtAl/. (4.3 MB ZIP). [file pbio.0020422.st001.zip › NielsenEtAl/html/1300.html]

AN4639.1.NCU00621.1.MG02874.1.FG08704.1


```
 CLUSTAL W (1.82) Multiple Sequence Alignments - Introns Inserted


Sequence 1: NCU00621.1	1414 aa
Sequence 2: FG08704.1	1330 aa
Sequence 3: MG02874.1	1582 aa
Sequence 4: AN4639.1	1431 aa
Alignment Length: 1606 aa
Number Identitical Residues: 616 aa
Alignment Score (without introns) 32866


MG02874.1 	MTAFTDLSCPHTARLTHKHLEPTAPDSASPQPPSARARAGQHRHHGPDSPSNATNHFHQF
NCU00621.1	------------------------------------------------------------
FG08704.1 	------------------------------------------------------------
AN4639.1  	--------------------------------------------------------MRDP
          	                                                          . 

MG02874.1 	AYPPSSIASGSSGAMPSRSHTTTTTTATLPAGPAAATMAASRLAQHNSPTPLPASVTSTN
NCU00621.1	-------------MSQVHPQRPEDGNQRVATNGTSHPAAHNQLPLRSNIQ----------
FG08704.1 	--------------MALRSDINGTSTPTAESNGVAS-AARPRPNGRSTSN----------
AN4639.1  	TVDMEQRNTSPHQASSLQAEAPRRTQKSVRVAFGPDLETHIPPRQRSPAP----------
          	:   ..  :..      :..              .   :      :.             

MG02874.1 	GAPARRPDLQTTGSSSSPNRRPSSAPSSKHS--ATAALANGDGFVANHMPVTVASRSSAA
NCU00621.1	-------RRMSDSSPAPLFRRPSSAPDDNNQTSPGTTLHGGQSDDDDHMPVARP------
FG08704.1 	-------DTTGNDEND---QRPLSAPGRRN-----GSIALETRDDIEHQQQTRP------
AN4639.1  	------ISNHHRSFTTVEHHKPPPARPTSSSGENTTPTEGNSRFTSDQDSSSSAR-----
          	            .      ::* .*     . .   .         ::   : .      

MG02874.1 	ADAASLSDRRPSSAKPKLLRSKSDFVLHSDDGYDSDPDNSDVDHHRRQGNNTKGKKISSW
NCU00621.1	-------------AKAPLTRSKSEYGLRLE---------------RESPIEQVDEEYHDW
FG08704.1 	-------------AKPLLLRSKSDYAPRPM---------------DEP--EPIEEDIPDW
AN4639.1  	-------------PTAALKRAKSDYGPRGG---------------FDKPVVVDNEDEEDF
          	             ... * *:**::  :                          :.  .:

MG02874.1 	GARHGFGDHYDSEEISHLVN0HAKLGFALRELLWLRLVPQNEGHSLPFFASYTHDKPMAT
NCU00621.1	GARHGFEDHYQSEEVIS---~-----------------------------------QLAN
FG08704.1 	GARHGFEDHYQSEDIIS---~-----------------------------------QLAN
AN4639.1  	AMRHGWQEEYTSSEYLK---~-----------------------------------VLHS
          	. ***: :.* *.:                                           : .

MG02874.1 	0NWYMYFTEKRHETTGKPKLAP--FEIQDWRMRDRLKTVSAAIAVCLNIGVEPPDQLKTN
NCU00621.1	0SWYMYFTDKRHETTGKPKTPA--FEIQDWRMRDRLKTVSAAIAVCLNIGVEPPDQLRTT
FG08704.1 	0NWYMYYTDKRHETTGKPKPPQ--YELQDWRQRDRLKTVSAALAVCLNIGVEPPDQLKTN
AN4639.1  	0NFYMYFTEKRHETNGFPRDPVGSWPSQDWRMKDRLKTVSAALAICLNIGVDPPDVVKTN
          	 .:***:*:*****.* *: . .::  **** :*********:*:******:*** ::*.

MG02874.1 	PGAKMEAWQDPTVTP---AQKALENIGKALQSQYETLAMRTRYKQYLDPSVEDTKKFCLS
NCU00621.1	PGARLEAWQDPTVPP---VSKALENIGKALQSQYETLAIRTRYKQYLDPSIEETKRFSVS
FG08704.1 	PGAKLEAWTDPTVPP---IQKALENIGKALQAQYETLAIRARYKQYLDPSVEETKKFCIS
AN4639.1  	PTAKLECWVDPTSTTGGGNNKLMEQIGKKLQEQYETLSLRTRYKQYLDPSVDETKKFCVS
          	* *::*.* *** ..... .* :*:*** ** *****::*:*********:::**:*.:*

MG02874.1 	LRKSAKDERVLLHYNGHGVPKPTSSGEIWVFNKNYTQYIPVSLYDLQHWLQAPTIFVWDC
NCU00621.1	LRRSAKDERVLFHYNGHGVPKPTASGEIWVFNKNYTQYIPVSLYDLQQWLQAPTIYVWDC
FG08704.1 	LRRNAKEERVLLHYNGHGVPKPTASGEIWVFNKNYTQYIPVSLYDLQHWLQAPTIFVWDC
AN4639.1  	LRRNAKDERVLFHYNGHGVPLPTQSGEIWVFNKNYTQYIPVSLYDLQQWLAGPSLFVFDV
          	**:.**:****:******** ** ***********************:** .*:::*:* 

MG02874.1 	SEAGNILSNYHKFVEKHEKEEDEAVLRDPNHYKANFRPYIHLAACSSSQNLPTNPLLPAD
NCU00621.1	SEAGNILTNYHRFVEKHEQEEEEAAQRDPNHEKINFRPYIHLAACNVKENLPTNPDLPAD
FG08704.1 	SEAGNILNNYHRFVEKHEEEEDEAAERDPNYTKTNFRPYIHLAACAVKENLPTNPLLPAD
AN4639.1  	SHAGNIVQNFHTFVEKHEKENLEQKRLDPNAIVQNYGDCIILAACQKNESLPTNPDLPAD
          	*.****: *:* ******:*: *    ***    *:   * ****  .:.***** ****

MG02874.1 	LFTCCLTTPIEMALWFFVLQNPLQTGLTPERAKKLPGRLQERRTPLGELNWIFTAITDTI
NCU00621.1	LFTCCLTTPIEMALWFFCLQNPLKTKISPERAKKLPGRLQERRTPLGELNWIFTAITDTI
FG08704.1 	LFTACLTTPIEMALWFFVLQNPLKTKVSPERAKKLPGRLQERRTPLGELNWIFTAITDSI
AN4639.1  	LFTCCLTTPIEIALRYFILQNPLQSNIRIDDFR-VPGRLQDRRSPLGELNWIFTAITDTI
          	***.*******:** :* *****:: :  :  : :*****:**:**************:*

MG02874.1 	AWTTLPRHLFRKFFRQDLMVAALFRNFLLAQRVMSVYGCTPQSFPELPDTRQHPLWESWD
NCU00621.1	AWTTLPRHLFRKFFRQDLMVAALFRNFLLAQRIMPVYNCHPQSYPELPDTRQHPLWESWD
FG08704.1 	AWTTLPRDLFRKFFRQDLMVAALFRNFLLAQRVMTVYGCHPQSYPKLPDTHQHPLWETWD
AN4639.1  	AWKTLPRALFKKLFRQDLMVAALFRNFLLSERIMRTYKCQPISSPELPETHNHPLWKSWD
          	**.**** **:*:****************::*:* .* * * * *:**:*::****::**

MG02874.1 	LAVDQALSQLPMLERKETEGIEYEYQNSTFFTEQLTAFDVYLTRGDALQQKPPEQLPVVL
NCU00621.1	LAVDLALAQLPALERKESEGIEYEYINSSFFAEQLTAFEIYLTRGDALSQKPPDQLPVVL
FG08704.1 	LAVDMALSQLPALEKKESEGYDYEYQNSSFFTEQLTAFDVYLTRGDAMAQKSPDQLPVVL
AN4639.1  	LAVEMVLAQLPALIDHEEGRRQYEYQHSTFFAEQLTAFEMYLSSG-PTEKSPPDQLPIVL
          	***: .*:*** *  :*    :*** :*:**:******::**: * .  :..*:***:**

MG02874.1 	QVLLSQQHRVRALILLGRFLDLGPWAVQLALSIGIYPYVLKLLQSAAAELKPVMVFIWTR
NCU00621.1	QVLLSQQHRVRALVLLARFLDLGPWAVQLALSIGIFPYVLKLLQSAAQDLKPVMVFIWTR
FG08704.1 	QVLLSQQHRVRALILLGRFLDLGPWSVQLALSIGIFPYVLKLLQSAAAELKPVMVFIWAR
AN4639.1  	QVLLSQAHRLRALILLSKFLDLGPWAVHLALSIGIFPYVVKLLQSAAQELKPVMVFIWAR
          	****** **:***:**.:*******:*:*******:***:******* :*********:*

MG02874.1 	VLAVDTSCQQDLIKDSGYQYFSNILKPSEGLPVANS---DEHKAMCAFILAMLCKGYKPG
NCU00621.1	VLAVDISCQQDLIKDSGYNYFAAIMKPQDTLSMVSVGFLDEHKAMCAFILAMLCKDYKPG
FG08704.1 	LIAVDLSCQQDLIKDSGYSYFAQILKPSEGLPVVDS---DEHKAMCAFILAMLCKDYKNG
AN4639.1  	IMAVDHTVQNDLLKDNGIHYFISILNPSSPIPVGNA---SEHRAMCAFIVSIFCKNYTQG
          	::*** : *:**:**.*  **  *::*.. :.: .    .**:******::::**.*. *

MG02874.1 	QVVCNQTDIMGYCLYHILNEDNPLLRQWSCLCISQLWYDLPEAKWRGIRDNAPARLSILT
NCU00621.1	QLVCNSTDIMTYCLYHIARPEGPLLKQWSCLCISQLWKDLPEGKWRGIRENATSKLSHLS
FG08704.1 	QMVCNQTDIMTYCLTHLQNEENPLLRQWACLCISQLWQDLPEAKWRGIRENAYVKLAYLV
AN4639.1  	QNVCLSSDLFESCLIHLMDVENPLLRQWSCLCISMLWSDFPEAKWMGIRCSAPMRLCELN
          	* ** .:*::  ** *:   :.***:**:***** ** *:**.** *** .*  :*. * 

MG02874.1 	KDGCSEVRAAVLYAMTTFLGIPDLTDEVARIEESIAWTVLDMANDGSPMVRKELLVFFSH
NCU00621.1	RDPNPEVRAAMAHAMTTFLGIPDLTDEVARIEESVAWTMLELATDGSPIVRKELLVFFSH
FG08704.1 	RDP---------------------------------------------------------
AN4639.1  	FDPVPEVRAAMLHALMTFLGIPDLTDQVAQIEESLALAVLPMASDGSVIVRKELLVFFST
          	 *  ..  ::   :  :  . .. :.. :  ..: : :    :...:    ..     : 

MG02874.1 	FILRYESKFLV~AAYEHLLEEKEYLAYPPHDDGRENKMGLHYSRPDKRGQDGSDRAKSQG
NCU00621.1	FVLRYESKFLV~AAFEQLSEEKDYLASPPPEDGQDHRMGLHYTRRENRERDGSIKPSAFG
FG08704.1 	---------CF1AAYEQLVEEKEYLIFPPQDDGQEHKMGLHYARPENRNKDGTIKPMAHG
AN4639.1  	FVKRYQNKFLV~AAYEELQDEKLNLLG---------KLGLDSSPPAETGTAGKPQP----
          	     ...  . **:*.* :**  *           ::**. :   :    *. :.    

MG02874.1 	LSHNTIIAAVWKHVNILSVDPHPEVQSHATTIVDYVHYTLLQSSLGSQSQSLMDDVGRRA
NCU00621.1	VAHDSIFAACWKHINILSVDPHPETQRDATIIVDYVHNALLQSPLTTQTQNLMDEILRRS
FG08704.1 	LSHNTVYMALWKLALVLSVDPHPEVQREATIVVDYVHHALLSSKVGAQTQLVMAEIQKRA
AN4639.1  	LSKNTTFGTIWKQLLILSVDPHPDIAQDAGIIVDYMHQLLLESPMAALTGRLRKEILDLS
          	:::::   : **   :*******:   .*  :***:*  **.* : : :  :  ::   :

MG02874.1 	RRQAARQQAQASQRNSINGGNSVGATTPLPSPG----LLRRTVSTLFSPFFPVDGPTTPT
NCU00621.1	RKVSRGDMSQRSAAAGTH----AAVAQPLPSPG----LLKRTASYLFGPLMGTLDPSSTS
FG08704.1 	TRMANRHMPNTSQRNNTIG---SSISQPLPSPG----LLRRTASLLFPSLVINEDKSRPT
AN4639.1  	NQVDQQSQVREKPNLKKAAPPPPPPTAPPKQEGYLSLSLRRTASVAAS--LKNLAFGGPS
          	 :       . .      .  .   : *  . *  :  *:**.*      .       .:

MG02874.1 	TPEPSPGLYRTSSVKSRKGAEPVVPVS------TQHNDHDVSHASYHVAREPVSAGFQDR
NCU00621.1	IPPLSPGLQRSGSRSSRKGPG-LDSAP------PEQNDQATSPANYHVANEPSCAGYKER
FG08704.1 	TPTLPR------SPSMKLVPN-QVPVP------PEQNDRTSAQALYNVAHEPYSGAFKER
AN4639.1  	QGESSQGSEASTSPQSRAPATPRGRAPPEWTRPPEVNDQLAPATTYQQAPVPTSRGFERR
          	    . .   : * . :  . .   .... : ..: **:  . : *: *  * . .:: *

MG02874.1 	DVTQAPKLPL~QSTLLAWSIEYFLEPQMKSSESEEPGSTEYNERLWRRSRNEGILNETQP
NCU00621.1	SMKDLPSLPL~VSTFLDWSIEYFREPQMKASEAEEPGSHEYNERLWRRSRNEAVLRETQP
FG08704.1 	DLTKPPTLPL~KSRFLEWSIEYFREPQMKPSEADEPGSTEYNERLWRRARNETIFRETQP
AN4639.1  	DSSVAPSIPL0----------YFREPQMKPNEPDEPGSADYNERLWRRSRNEKIISETQP
          	. .  *.:**           ** *****..*.:**** :********:*** :: ****

MG02874.1 	QKLQAGRHKWNNQTGIFNTHAQPSKLNFHQFEDHVAVADDGNTIQ~IWDWKNQGRLSRFS
NCU00621.1	QKTHARTHKWNDQIGVINNGSQPAKLSFHQFENYVAVADDGNTVN~VWDWKQGTRKSRFS
FG08704.1 	LKQQAGSHKWNNQLGIVNNGAQPAKMTFHQFEDHLAVADEGNTVY~VWDWKKQGRLSRFS
AN4639.1  	LKGKAGSSRWDNSIALLSNATQPLKMCFHQFEDHIAVADDRDTIA2IWDWQNHKRLNRFS
          	 * :*   :*::. .:... :** *: *****:::****: :*:  :***::  * .***

MG02874.1 	NGNPEGSRISDMKFINEEDQALLMTGSSDGVIRIYRNYDSHDDVELSSSWRALTDMVPSN
NCU00621.1	NGNPEGSKISDMKFINEDDQALLMTGSSDGVIRVYRNYDSNENIELASAWRALTHMVPSN
FG08704.1 	NGNPEGSKISDMKFINEDDQAMLLTGSSDGVIRVYRNYESDRAIELASSWRALTHMVPSN
AN4639.1  	NGNPLGSKINEVRYINEDDQALLMTGSSDGVLKVFRNYESAKDVEIVTAFRALPELIPSN
          	**** **:*.::::***:***:*:*******::::***:*   :*: :::***..::***

MG02874.1 	VNSGMVFDWQQVNGTTLVAGDVRVIRVWSAGQEACVT~DIPARSGSCVTSLTSDQMTGNV
NCU00621.1	VNSGMVFDWQQVKGQVLVAGDERVIRIWNAGHEMCTH~EIPARSGSCVTSLTSDQMTGNI
FG08704.1 	VNSGMVFDWQQVTGRVLVAGDVRVIRVWYAAYETCVM~DIPARSGSCVTSLTSDQMTGNM
AN4639.1  	RNAGLVLDWQQGQGKALVAGDVKVIRVWNAATEVCTT0DIPARSGSCITSLTSDQVAGNI
          	 *:*:*:****  * .***** :***:* *. * *.  :********:*******::**:

MG02874.1 	FVAGFGDGAVRVFDIRLRPQDSMIRKWKDETDRVWIKTVAMQRGGQRELMSASRNGKVKL
NCU00621.1	FVAGFGDGAIRVFDTRMKPQESMVRKWKDEK-RQWVRSVHMQRGGQRELLSASRNGKIRL
FG08704.1 	FVAGFGDGAVRVFDTRNRPQESMVRKWKDESDRQWVKSVHMQRGGQRELVSASRNGKVKI
AN4639.1  	FVAGFGDGAVRVFDQRLKPTASMVKVWREHK--QWITNVHMQRGGLRELISGSRNGEIRL
          	*********:**** * :*  **:: *::..   *: .* ***** ***:*.****::::

MG02874.1 	WDIRMDKPLSVFQTTKETLRTASMHEHLPVFSV2GTSAHTVKVFNLDGKELSQMEPYSSF
NCU00621.1	WDIRMDQPLKSFQTTKDVLRTASTHEHLPVFAV2GTSAHLVKVFDFDGHELTRIEPYSSF
FG08704.1 	WDIRMDKPLHSFQTTRDTLRTASTHEHLPVFAV2GTSAHTVKVFNLDGHELSSVEPYSSF
AN4639.1  	WDLRMDNPISTIYGTRDTLRTLSVHEHAPVFMV2GTNRHEVKTYNVDGTHLSTFEPYSSF
          	**:***:*:  :  *::.*** * *** *** * **. * **.::.** .*: .******

MG02874.1 	LQ-NRGSPISATAFHPHHAILGCAARGDHHVNL~YQCSG~AR~IPQADEKGDA---
NCU00621.1	LQGHKGTPISATAFHPHRMVLGCAARGDYHVNL~FACGN~EK~VGPF---------
FG08704.1 	LQQNRSSAISATAFHPHRPILGCAARGDHHINL~FTCEK~SE~SMSSN--------
AN4639.1  	LHHNRSSPISSTAFHPHRTLFACAALNDNHINL0HGRQF0VC1PALVKQSNGLEYL
          	*: ::.:.**:******: ::.*** .* *:** .            ..... .
```
